# Skin Ultrasound Findings of *Mycobacterium abscessus* After Cosmetic Procedures: The Four Stages of Evolution

**DOI:** 10.1111/jocd.70466

**Published:** 2025-10-07

**Authors:** Leila Riedel, Andrezza Sarli, Daniella Prudente Martins, Maria Claudia Almeida Issa

**Affiliations:** ^1^ Private Office Niterói Rio de Janeiro Brazil; ^2^ Private Office São Paulo São Paulo Brazil; ^3^ Universidade Federal da Bahia Salvador Bahia Brazil; ^4^ Department of Internal Medicine, Dermatology Service, Hospital Universitário Antonio Pedro, Universidade Federal Fluminense Niteroi Rio de Janeiro Brazil

To the Editor,

1

High‐frequency cutaneous ultrasound (HFUS) is a well‐established tool for diagnosing, guiding, and following up on aesthetic procedures and related complications [1, 2]. Skin US is mainly relevant in vascular obstruction, inflammatory and non‐inflammatory nodules, and abscesses [3]. *Staphylococcus aureus* , *Streptococcus pyogenes* , and non‐tuberculous mycobacteria (NTM) are the most reported pathogens associated with post‐procedural skin infections [4]. Among the NTM, *Mycobacterium abscessus* is the most significant species, particularly after liposuction and mammoplasty [5], probably due to the improper cleaning.

HFUS plays a crucial role in supporting early diagnostic suspicion and treatment, particularly in suspected cases of NTM infection. In the early stages, cutaneous lesions caused by NTM may clinically resemble common bacterial abscesses, presenting with inflammatory signs such as erythema, edema, and firm nodules draining purulent content. However, HFUS reveals distinct features depending on the disease stage. We propose four stages of evolution: Stage I—Granulomatous Phase: Hypoechoic nodular lesion located in the dermis or hypodermis, with increased echogenicity of the surrounding subcutaneous tissue (suggestive of panniculitis) and moderate intralesional and peripheral vascularity (Figure 1a). Stage II—Liquefaction Phase: Heterogeneous iso/hypoechoic collection with nonmobile internal content. Anechoic peripheral zones suggest centripetal liquefaction. Adjacent panniculitis is evident as increased echogenicity and marked vascularity within and around the lesion. These findings support the presence of residual fat lobules within the collection, likely undergoing inflammatory liquefaction (Figure 1b). Stage III—Tunnelization Phase: Interconnected hypoechoic linear tracts extending toward the dermis or skin surface, associated with heterogeneous fluid content and peripheral vascularity. Echogenic subcutaneous fat persists, indicating ongoing panniculitis (Figure 1c). Stage IV—Mixed Phase: Coexistence of linear tracts and nodular lesions, with persistent heterogeneous debris and thickened, irregular walls. Vascular signals are centrally and peripherally present (Figure 1d and Table 1).

**FIGURE 1:**
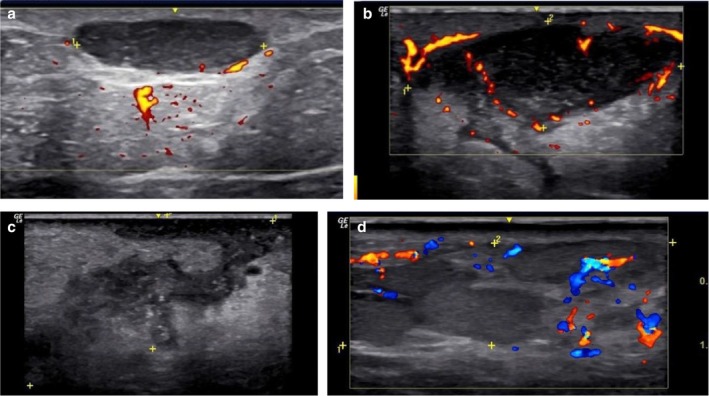
(a) Stage I (granulomatous phase): Hypoechoic nodular area with increased echogenicity of the subcutaneous tissue (panniculitis) and moderate intralesional and peripheral vascularity on Doppler. (b) Stage II (liquefaction phase): Hypoechoic heterogeneous collection with internal echoes, increased echogenicity of the subcutaneous tissue (panniculitis), and central and peripheral vascularity. (c) Stage III (tunnelization phase): Interconnected hypoechoic sinus tracts with heterogeneous internal echoes, increased echogenicity of the subcutaneous tissue (panniculitis), and predominantly peripheral vascularity. (d) Stage IV (mixed phase): Interconnected hypoechoic sinus tracts with heterogeneous internal echoes, increased echogenicity of the subcutaneous tissue (panniculitis), and a hypoechoic nodular.

**TABLE 1:** Clinical aspects and HFUS findings with schematic drawings of each stage.

	Clinical aspects	Ultrasound findings	Schematic drawings
Stage I	Erythema, oedema, and hard nodules	Hypoechoic nodular area with increased echogenicity of the subcutaneous tissue (panniculitis) and moderate intralesional and peripheral vascularity on Doppler	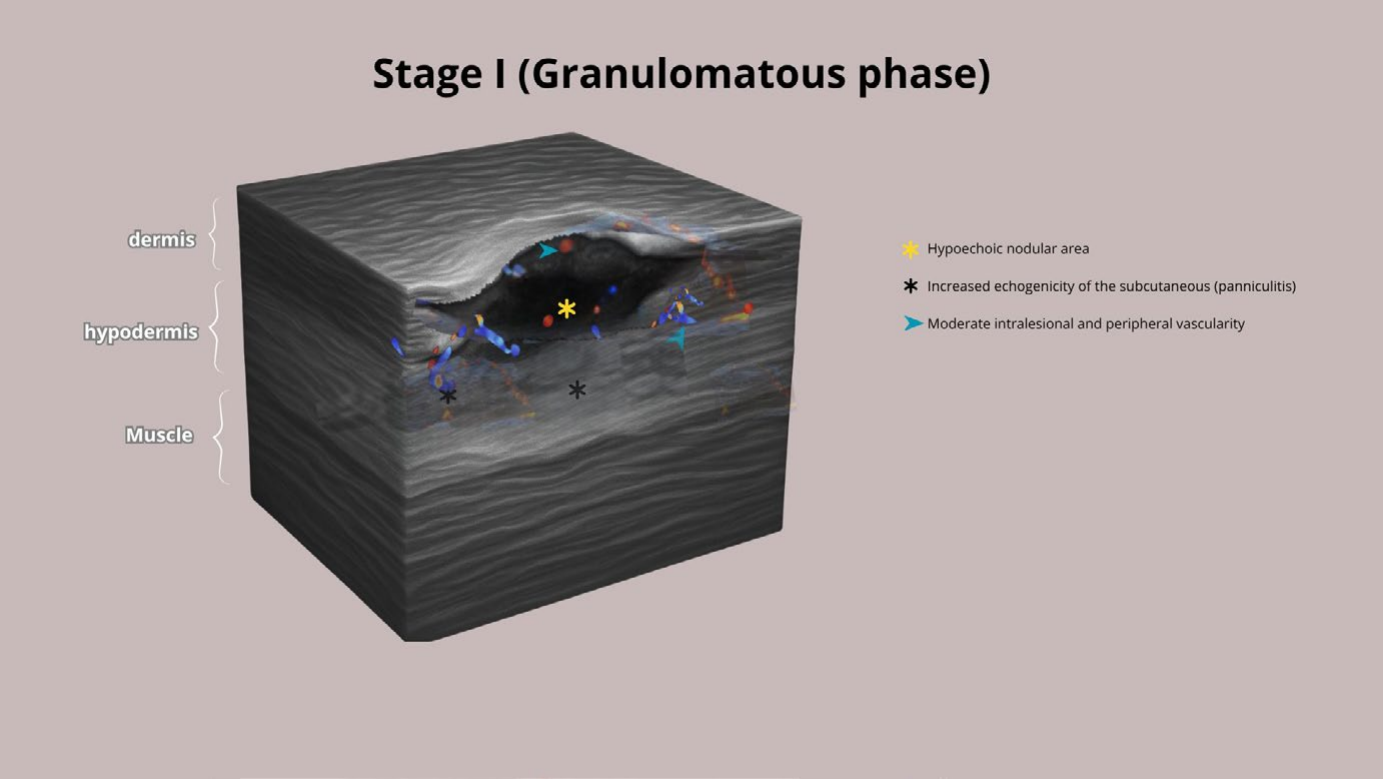
Stage II	Erythema, edema and softened nodules	Hypoechoic heterogeneous collection with internal echoes, increased echogenicity of the subcutaneous tissue (panniculitis), and central and peripheral vascularity	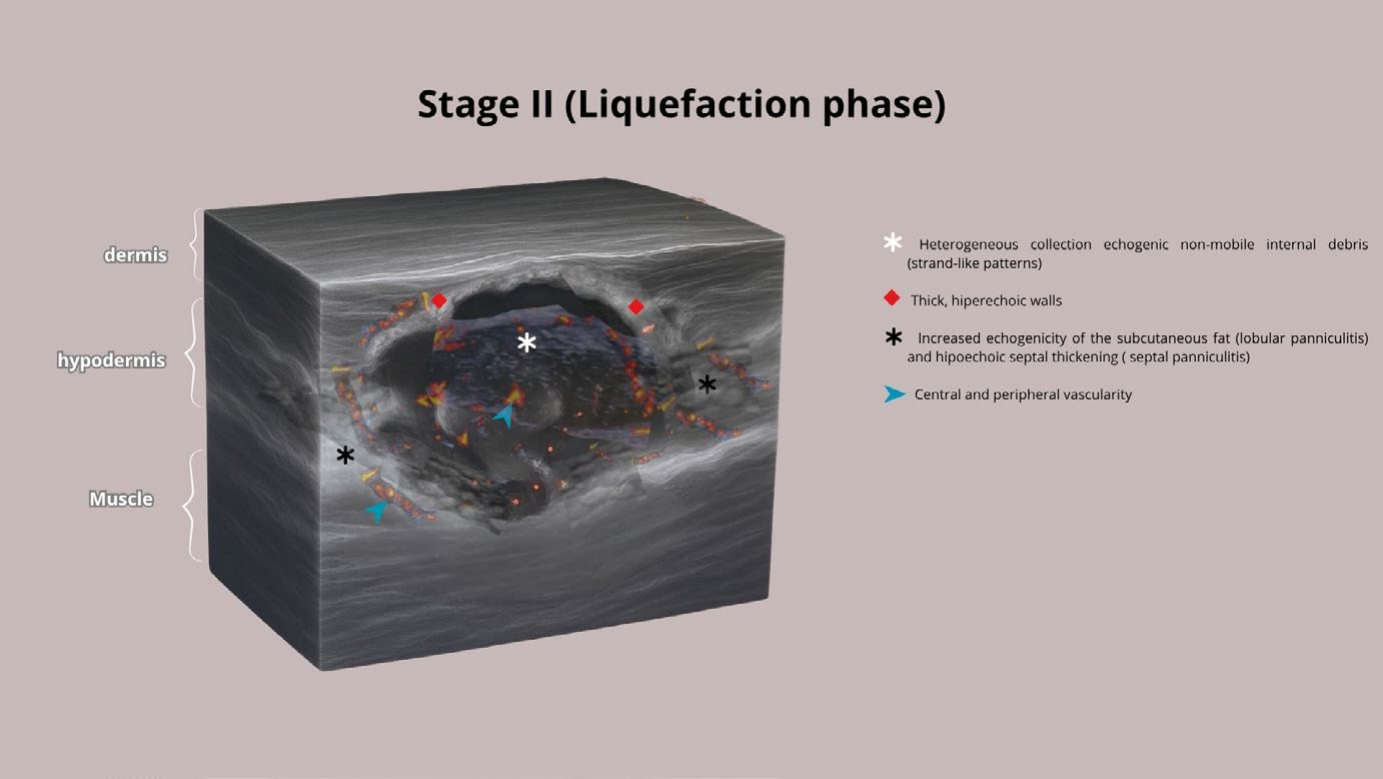
Stage III	Erythema, edema, softened nodules, fistulas, and crusts	Interconnected hypoechoic sinus tracts with heterogeneous internal echoes, increased echogenicity of the subcutaneous tissue (panniculitis), and predominantly peripheral vascularity	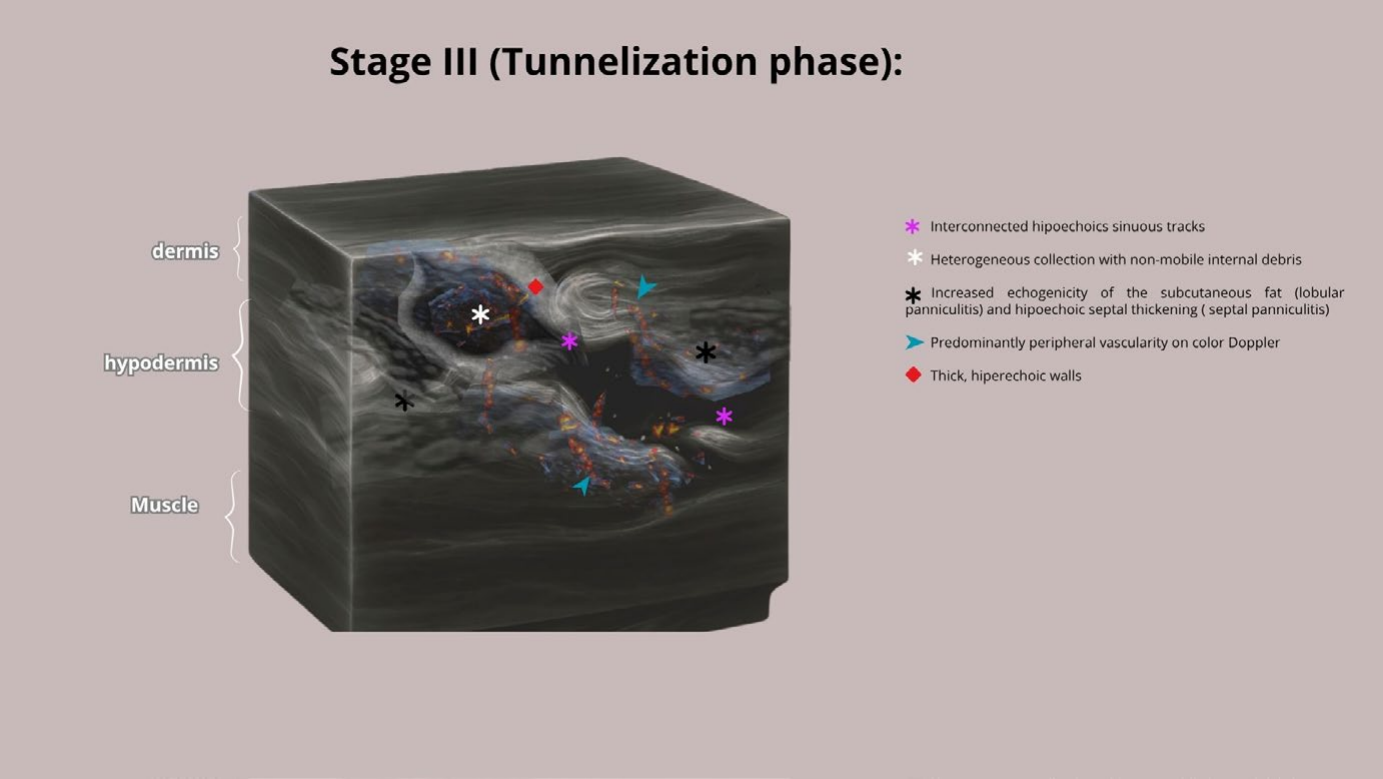
Stage IV	Erythema, edema, hard and softened nodules, fistulas, and crusts	Interconnected hypoechoic sinus tracts with heterogeneous internal echoes, increased echogenicity of the subcutaneous tissue (panniculitis), and a hypoechoic nodular	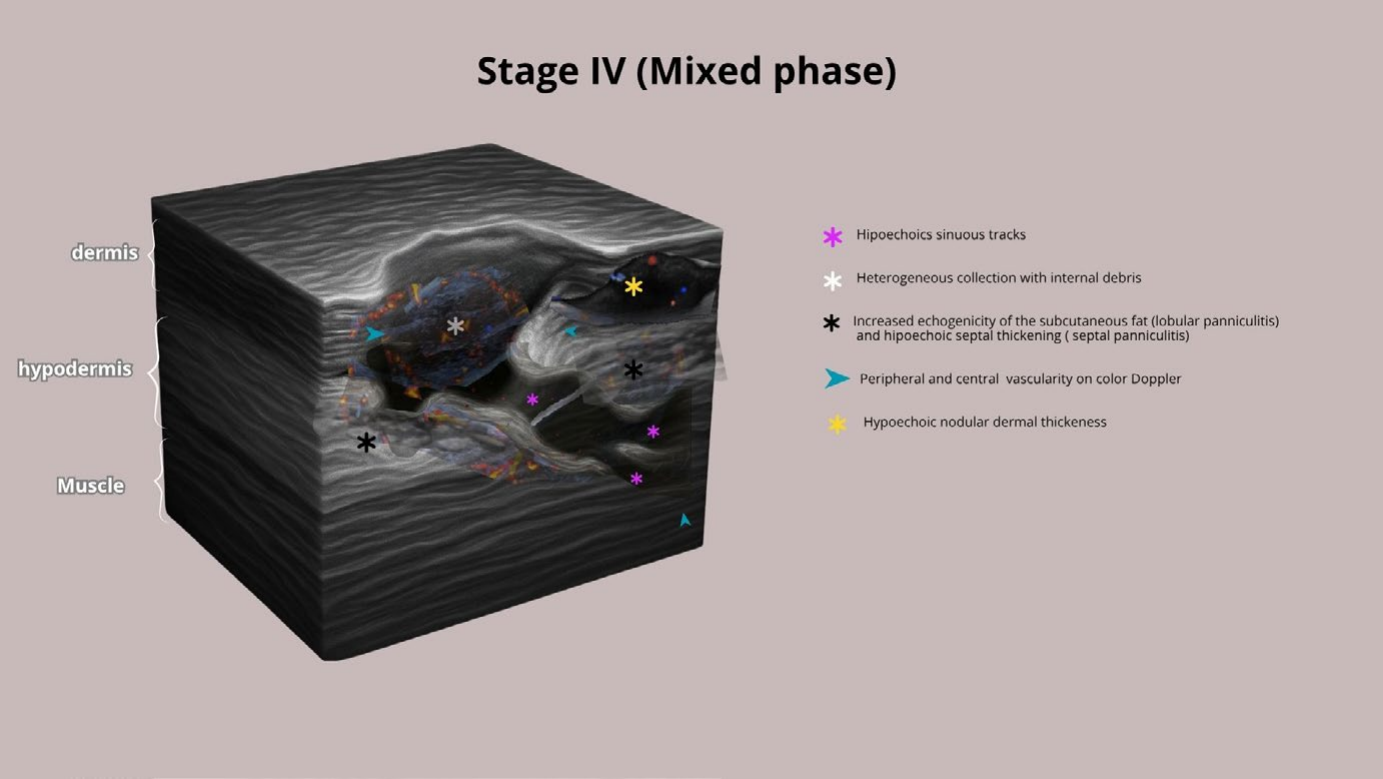

HFUS consistently reveals relevant differences between pyogenic and NTM abscesses (Table 2). Pyogenic abscesses are usually solitary, well‐circumscribed, and unilocular, with anechoic fluid and mobile internal debris. They exhibit thin, regular walls, intense peripheral vascularity, and no internal flow (Figure 2a–c). Perilesional panniculitis is common, reflected by increased echogenicity of the adjacent fat. In contrast, NTM‐related abscesses tend to be multiple and initially present as a dermal thickening. The collections are multiloculated, with nonmobile, reticulated internal content and a peripheral‐to‐central liquefaction pattern. A central echogenic fat island may remain in early stages, consistent with isolated panniculitis. Both peripheral and central vascularity are commonly observed due to inflammation of the trapped fat. As the infection progresses, fistulous tracts toward the skin surface develop, and the walls become thick and irregular.

**TABLE 2:** Comparative findings of pyogenic abscess vs. NTM abscess in HFUS.

HFUS findings	Pyogenic abscess	NTM abscess
Number and pattern of lesions	Typically, single, unilocular Subcutaneous lesion since the beginning	Multiple Dermal lesion in the initial phase
Perilesional tissue	Increased echogenicity of the surrounding subcutaneous tissue	Increased echogenicity of the surrounding subcutaneous tissue
Internal content	Predominantly hypoechoic Mobile echoes	Heterogeneous echogenicity Nonmobile reticulated central content—“fat island”
Liquefaction pattern	Diffuse and heterogeneous liquefaction	Peripheral to central liquefaction Subcutaneous tissue may persist as “fat islands”
Lesion wall	Thin, regular	Thick, irregular
Doppler vascularization	Intense peripheral flow No central vessels	Peripheral and central vessels
Evolution	No development of fistulous tracts	Development of fistulous tracts

**FIGURE 2:**
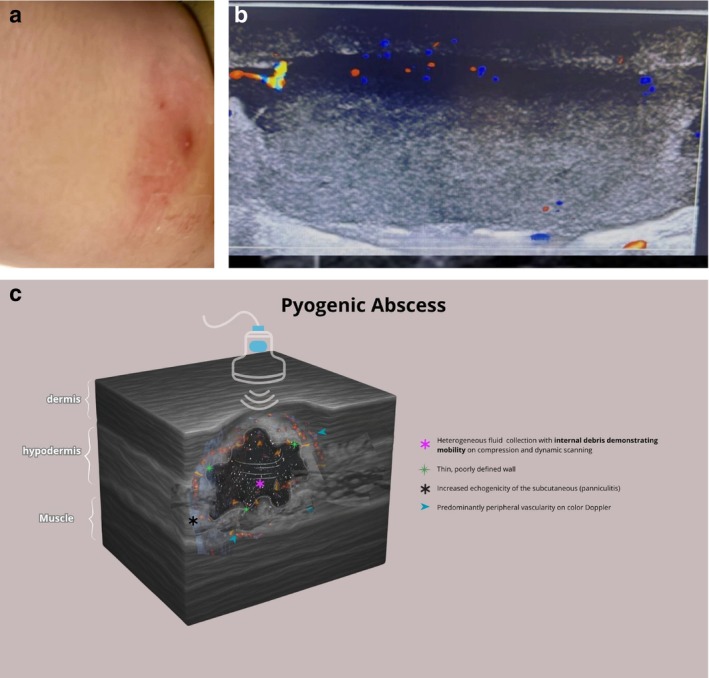
(a) Clinical aspect of a pyogenic abscess on the thigh. (b) Pyogenic abscess's HFUS findings: Solitary, well‐circumscribed, and unilocular, with anechoic fluid and mobile internal debris, thin and regular walls, intense peripheral vascularity, and no internal flow. (c) Pyogenic abscess's schematic drawing: heterogeneous fluid collection with internal debris demonstrating mobility on compression. Thin, poorly defined wall. Increased echogenicity of the subcutaneous (panniculitis). Predominantly peripheral vascularity on color Doppler.

Patient 1: Female, 42 years old, underwent submental liposuction and facial liposculpture. Two weeks later, she had a fever with facial edema, erythema, and multiple painful erythematous cutaneous nodules followed by fistula and crust formation on the middle and lower face (Figure 3). Patient 2: Female, 45 years old, received a laser lipolysis treatment in the submental area with facial liposculpture. After 10 days, she developed erythematous nodules, fistulas, and crusts on the middle and lower face (Figure 4). Patient 3: Female, 29 years old, underwent breast augmentation with silicone implants. One month after the procedure, she developed erythema, edema, and purulent discharge at the surgical scar (Figure 5). Surgical explantation of the implant was required. Patient 4: Male underwent enzyme lipolysis in the abdominal region. One month later, he developed erythematoviolaceous nodules followed by fistulization and crust formation (Figure 6). In all cases, HFUS findings varied according to the proposed stages. Tissue culture confirmed *M. abscessus* subsp. *abscessus* via PCR‐restriction enzyme analysis (PRA). Targeted therapy was implemented.

**FIGURE 3:**
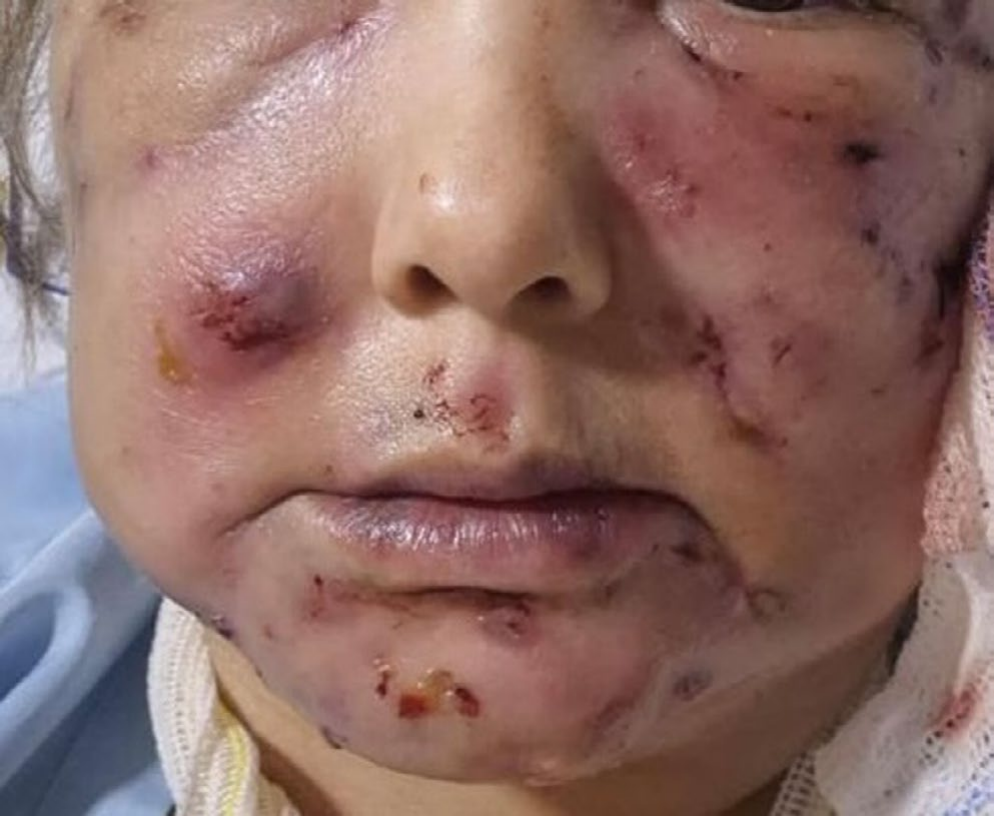
Facial edema with multiple erythematous nodules and cutaneous fistulous opening to the skin with crusts on the middle and lower face.

**FIGURE 4:**
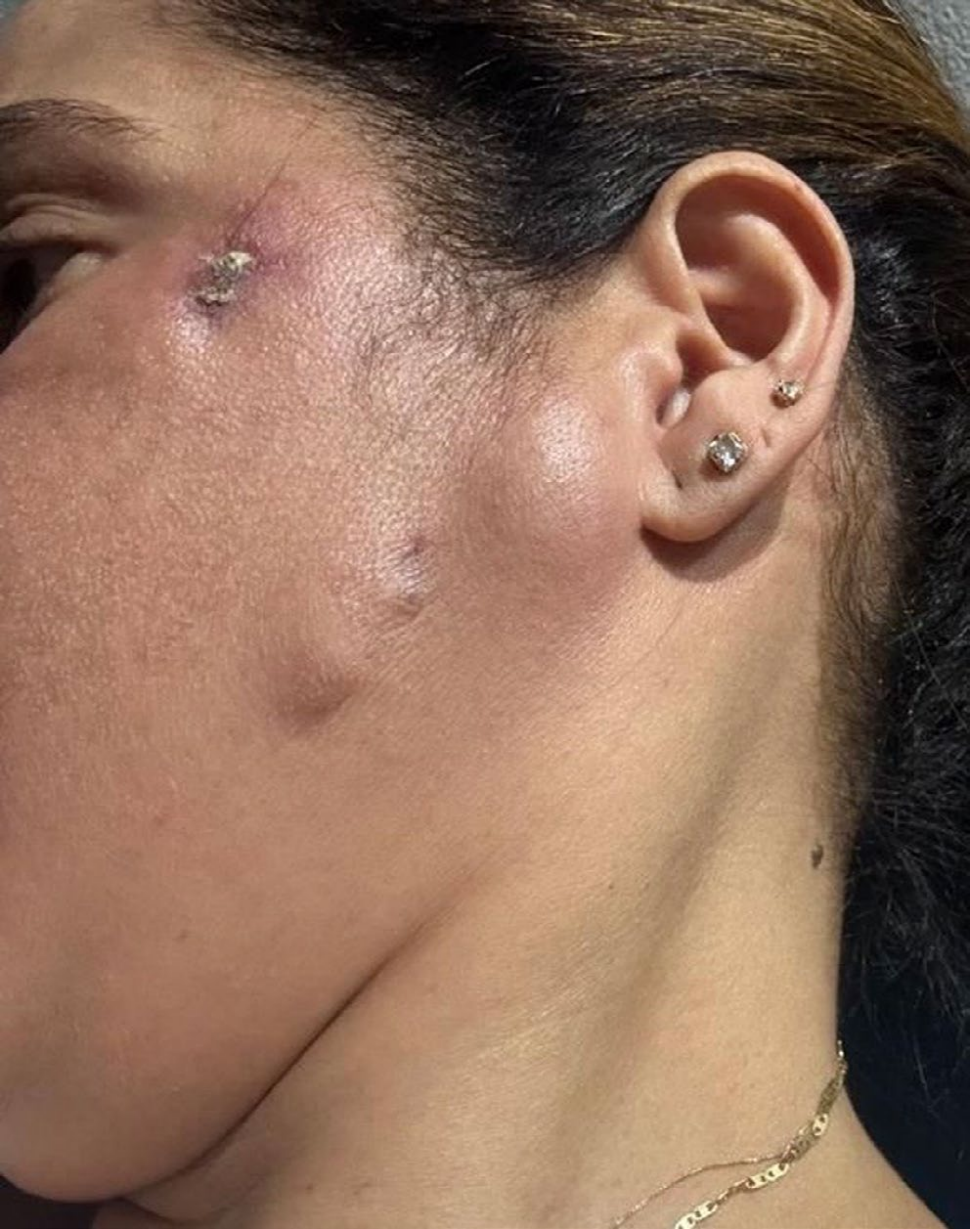
Facial edema with multiple erythematous nodules and cutaneous fistulous opening to the skin with crusts on the middle and lower face.

**FIGURE 5:**
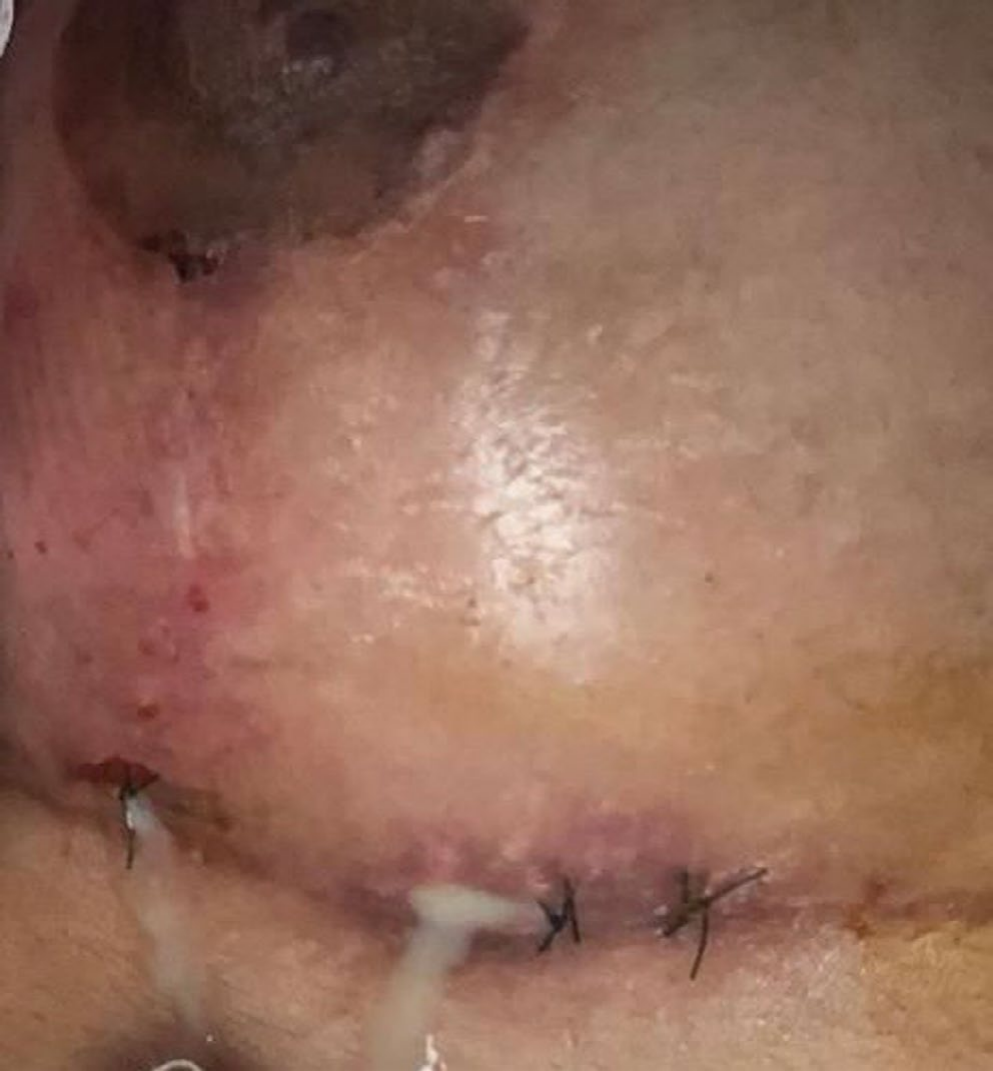
Erythema and edema around the breast surgical scar with purulent discharge.

**FIGURE 6:**
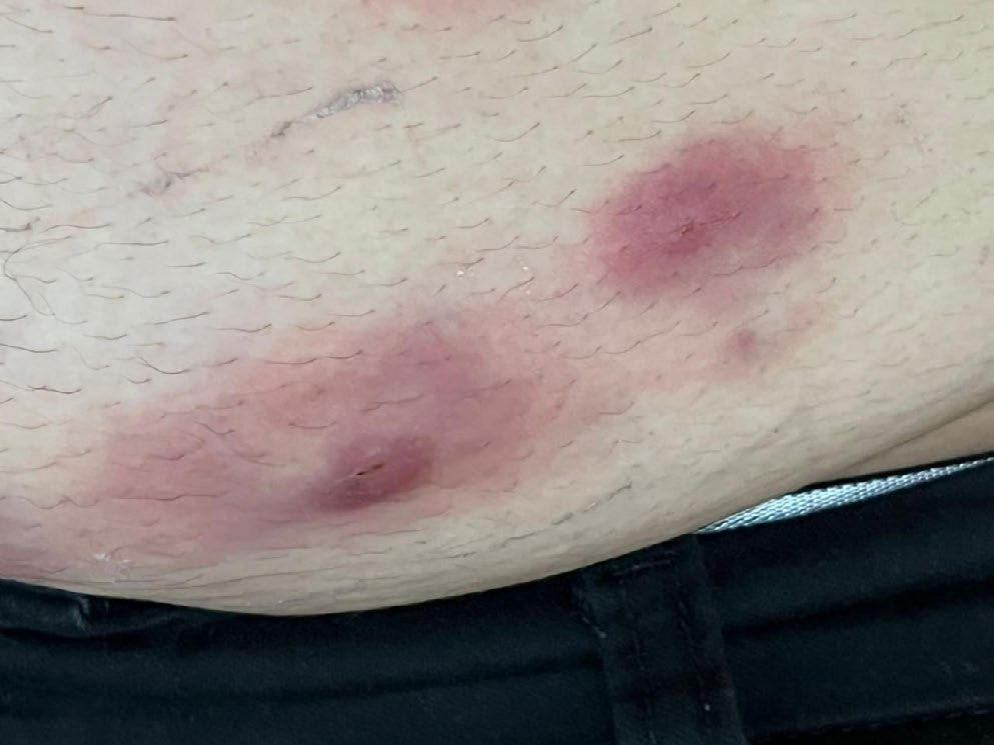
Erythematoviolaceous nodular lesions on the abdomen.

*M. abscessus* is classified as a rapidly growing mycobacterium. This classification is based on its ability to form visible colonies on solid media within 7 days [6]. *M. abscessus* is part of the NTM group and is known for its adaptability and virulence, contributing to its pathogenicity in humans. It comprises three subspecies: *M. abscessus* subsp. *abscessus*, *M. abscessus* subsp. *massiliense*, and *M. abscessus* subsp. *bolletii*. Intrabacterial lipid inclusions during infection may contribute to its persistence and survival in lipid‐rich environments. It suggests that *M. abscessus* may be able to utilize host‐derived lipids for energy and carbon storage, potentially influencing its growth dynamics in lipid‐rich tissues. This ability to adapt and persist in lipid‐rich environments is related to cosmetic procedures involving fat tissue, as in our cases. The clinical evolution of our patients, in whom lesions appeared after 30 days, corroborates what has been reported in the literature [5]. *M. abscessus* pathogen should be considered when evaluating patients who have undergone cosmetic procedures, particularly liposuction, followed or not by liposculpture. Differentiating pyogenic abscesses from NTM abscesses is critical for patients' outcomes, and the HFUS findings should be looked at to support the clinical hypothesis. Our proposal may contribute to doctors' future diagnoses and early, specific treatment.

## Consent

Informed consent was obtained from all individual participants included in the study.

## Conflicts of Interest

The authors declare no conflicts of interest.

## Data Availability

The data that support the findings of this study are available on request from the corresponding author. The data are not publicly available due to privacy or ethical restrictions.
